# Pes Anserine Ganglion Cyst as a Rare Cause of Chronic Medial Knee Pain: A Case Report

**DOI:** 10.7759/cureus.102249

**Published:** 2026-01-25

**Authors:** Alex Abouafech, Margaret Ferrara, Richard L Snyder

**Affiliations:** 1 School of Medicine, Lake Erie College of Osteopathic Medicine, Bradenton, USA; 2 Department of Orthopedic Surgery, Southeast Orthopedic Specialists, Jacksonville, USA

**Keywords:** arthroscopic excision, ganglion cyst, magnetic resonance imaging, medial knee pain, pes anserine bursa

## Abstract

Ganglion cysts are benign mucin-filled pseudocysts most commonly encountered in the wrist and hand, while intra-articular ganglion cysts of the knee are rare, particularly those arising near the pes anserine bursa. Their nonspecific presentation often overlaps with more common causes of medial knee pain, leading to diagnostic delay. We report the case of a 43-year-old woman who presented with progressively worsening, atraumatic medial knee pain over several months with a largely unremarkable physical examination. Plain radiographs were normal, while magnetic resonance imaging demonstrated a well-circumscribed, multilobulated cystic lesion adjacent to the pes anserine bursa without associated meniscal or ligamentous pathology. Due to persistent symptoms despite conservative management, arthroscopic excision was performed, confirming a ganglion cyst on gross pathology. The patient experienced complete resolution of symptoms without recurrence at the six-week follow-up. This case highlights the importance of considering pes anserine ganglion cysts in the differential diagnosis of chronic medial knee pain and underscores the critical role of MRI in accurate diagnosis and surgical planning.

## Introduction

Ganglion cysts are benign, mucin-filled pseudocysts originating from myxoid degeneration of connective tissue. They appear to originate from the translocation of synovial cells or the extravasation of synovial fluid caused by chronic injury or repetitive irritation [1, 2]. The walls of the cysts are composed of fibrous tissue that adheres to adjacent tendon sheaths, ligaments, and joint capsules [3, 4].

Ganglion cysts are frequently found on the dorsal wrist, volar wrist, and digital flexor tendon sheaths [3, 4]. However, less commonly, they can be found around the cruciate ligaments, menisci, and tibiofibular joint [2]. They are commonly discovered on imaging or arthroscopy, often incidentally, appearing as a well-defined mass with the usual tail sign on ultrasound [2]. MRI is the preferred modality for confirming the cysts [5]. 

Although distal upper extremity intra-articular ganglion cysts are frequently encountered, cysts originating around the pes anserine bursa are atypical [3]. They typically present with localized tenderness and swelling distal and medial to the tibial plateau at the insertion of the conjoined tendons: sartorius, gracilis, and semitendinosus [6]. Diagnosis is often prolonged as their clinical presentations are frequently superimposed with more common pathologies such as pes anserine bursitis, degenerative joint disease, or meniscal tears [6]. 

We present a case of a 43-year-old female patient with chronic medial knee pain caused by a ganglion cyst arising near the pes anserine bursa, emphasizing the importance of MRI in accurate diagnosis and surgical excisional planning.

## Case presentation

A 43-year-old woman with no significant past medical history presented for evaluation of right medial knee pain that progressively worsened over two to three months. The pain began insidiously without trauma, swelling, or instability. It was exacerbated by activity, prolonged standing, and stair climbing, and partially relieved by rest. The patient denied locking, giving way, redness, or systemic symptoms. There was no prior surgery, arthritis, or infection involving the knee. Initial management included rest, activity modification, alternating ice and heat therapy, and over-the-counter nonsteroidal anti-inflammatory drugs (NSAIDs), with minimal relief. She denied any constitutional symptoms such as fever, weight loss, or malaise. The remainder of her review of systems was unremarkable.

Upon physical exam, the patient appeared healthy and well nourished, in no acute distress. On inspection, there was no deformity, effusion, erythema, or evidence of muscle atrophy. Range of motion was full, from 0° of extension to 140° of flexion. Palpation revealed mild tenderness along the medial joint line and the pes anserine region, without palpable crepitus or warmth. Stability testing demonstrated negative Lachman, anterior and posterior drawer, pivot shift, and varus/valgus stress tests. Special testing with McMurray and Apley compression maneuvers elicited mild medial discomfort without a definitive click. The neurovascular examination was intact, with preserved sensation, 2+ distal pulses, brisk capillary refill, and a normal gait.

**Figure 1:**
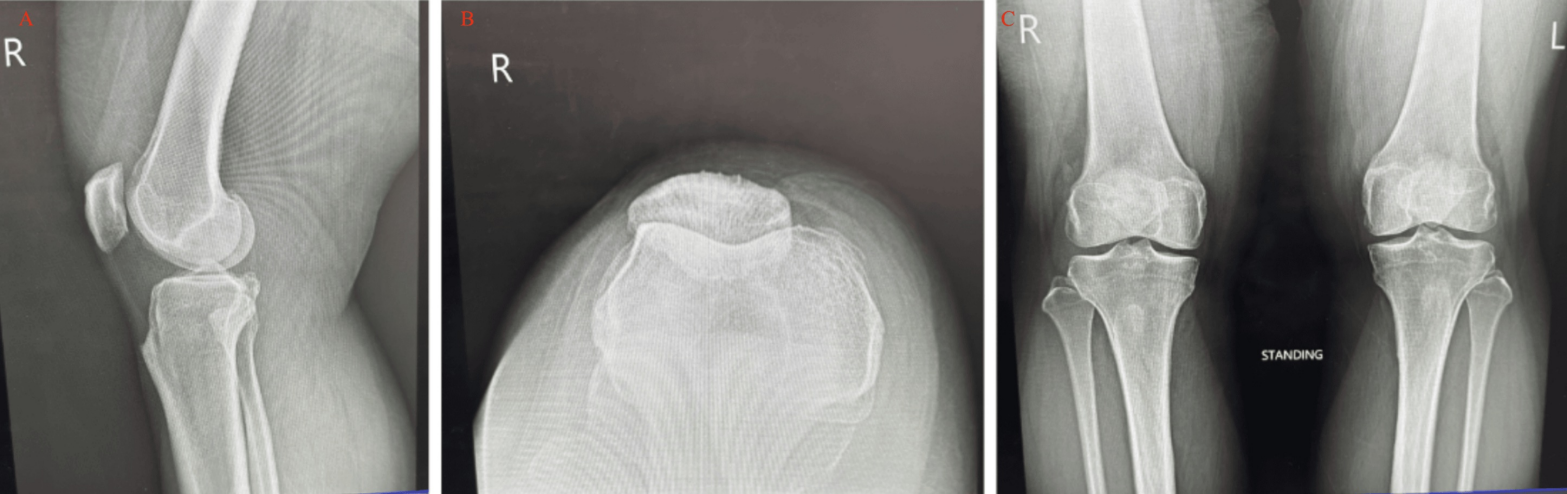
Plain radiographs of the right knee with a lateral view (A), sunrise view (B), and then bilateral anterior-posterior view (C). No joint space degeneration or osseous abnormalities present. Slight patellofemoral syndrome is present, as indicated by the lateral tracking of the patella on the sunrise view.

Imaging was ordered to further evaluate the cause of the patient’s persistent medial knee pain despite a largely normal physical examination. Plain radiographs of the right knee were normal with preserved joint spaces and no osseous abnormalities, as shown in Figure 1. MRI revealed a well-circumscribed, multilobulated cystic lesion measuring approximately 1.5 × 1.0 cm along the medial joint line, adjacent to the meniscocapsular junction and pes anserine bursa. The lesion was hypointense on T1-weighted and hyperintense on T2-weighted sequences, with no enhancement or evidence of meniscal tear. Although the patient was willing to share the MRI report, they declined to share the MRI scans.

**Figure 2:**
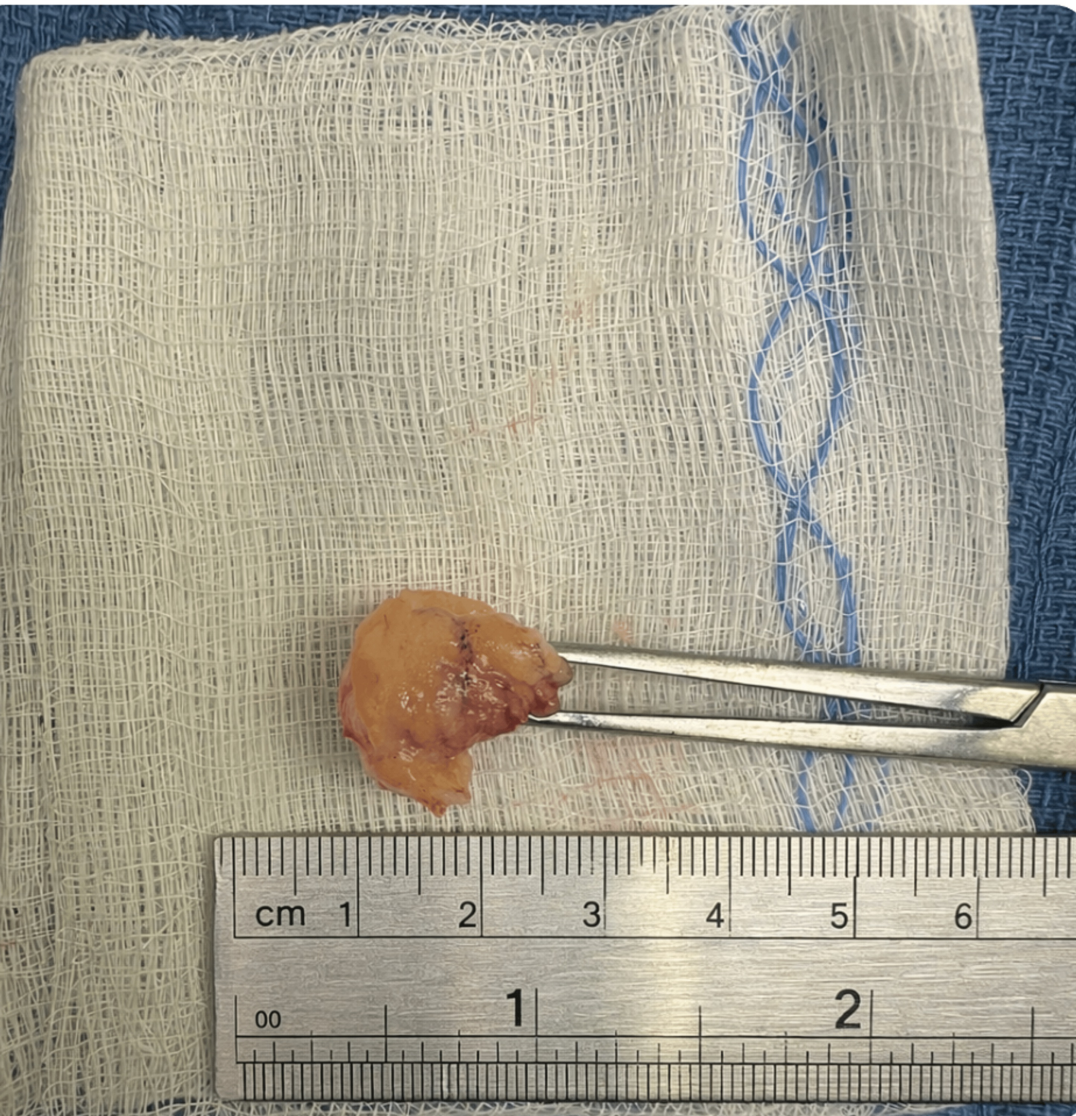
The excised cyst is pictured above. Grossly, the lesion measured approximately 1.8 × 1.3 × 0.9 cm.

After a multidisciplinary discussion and review of imaging, the patient was counseled on treatment options, including continued conservative management versus arthroscopic excision. Given the persistence of symptoms and cyst size, surgical management was recommended. The risks of surgery, such as bleeding, infection, neurovascular injury, stiffness, and deep venous thrombosis, were discussed in detail. Arthroscopic excision of the cyst with inspection of the menisci and capsule was performed, and the cyst was removed as shown in Figure 2.

Postoperative rehabilitation included early range-of-motion exercises and a gradual return to activity. At the six-week follow-up, the patient reported complete resolution of pain and no recurrence of swelling or mechanical symptoms.

## Discussion

Ganglion cysts of the knee are relatively uncommon, especially near the pes anserine bursa. Most intra-articular cysts arise on the anterior cruciate ligament, posterior cruciate ligament, and menisci, whereas extra-articular ganglion cysts commonly originate on the proximal tibiofibular joint [7]. Imaging of the ganglion cysts can be seen on ultrasound, arthrography, and MRI. 

The exact underlying pathogenesis of soft tissue ganglion cysts remains unclear. The cysts are outpouches of the synovial joint capsule, filled with mucinous fluid, and are attached to adjacent tissues through a thin stalk. The stalk provides a link whereby the contents pass between the cyst and joint capsule, causing the size to often fluctuate [3, 4]. The cyst walls lack a true epithelial lining, possibly due to mucoid degeneration caused by repetitive injury, chronic irritation, or chronic ischemia [2]. 

The pes anserine bursa is located 2 cm distal to the medial side of the tibial plateau, which is beneath the conjoined tendons of the sartorius, gracilis, and semitendinosus muscles. The location of the pes anserine bursa can produce cysts through chronic inflammation from overuse injury and buildup of fluid within the bursa [8]. 

Most patients with ganglion cysts are asymptomatic, in that only about 10% are responsible for symptomatic knee complaints [7]. Patients presenting with a ganglion cyst near the pes anserine bursa often report pain along the medial joint line and decreased range of motion. The pain is commonly chronic and intermittent, often exacerbated during flexion of the knee [1]. Symptoms frequently overlap with pes anserine bursitis or meniscal tear, resulting in delayed diagnosis without proper imaging. 

Diagnosis of ganglion cysts of the pes anserine bursa is done through MRI. Although ultrasounds and arthrography can be used, they are not the gold standard diagnostic modalities because of inconsistency in the identification of cysts smaller than <5 mm and invasiveness in the latter [1]. Thus, MRI is the standard of care in diagnosing ganglion cysts by providing the most accurate evaluation of the cyst’s size, morphology, and intra-articular location to adjacent anatomic structures [7]. Previous MRI studies have shown a 0.4% to 1.3% prevalence of intra-articular ganglion cysts of the knee [9,10].

Management of the cysts is dependent on the symptomatology the patient is experiencing. For the asymptomatic patients, conservative treatment through observation is appropriate as the size may reduce spontaneously [7]. If symptoms of pain, decreased range of motion, or swelling emerge during observation, treatment options can be considered. 

Ultrasound-guided aspiration of the cyst with a corticosteroid injection is a minimally invasive procedure, and 90% of patients report immediate symptom improvement [11]. However, aspiration alone commonly has high recurrence rates. Surgical excision is often the preferred treatment, as it offers a 7.5% recurrence rate, as compared to a 35.7% recurrence rate for ultrasound-guided aspiration [12]. Arthroscopic excision provides direct visualization of the joint cavity to ensure complete cyst and cyst wall removal, while also providing tissue for histologic confirmation [7].

This case exemplifies the supported diagnosis of an extra-articular ganglion cyst of the pes anserine. Ganglion cysts should be considered in the differential diagnosis in the setting of chronic medial knee pain in the absence of intra-articular pathology and normal ligamentous anatomy.

## Conclusions

Ganglion cysts arising near the pes anserine bursa represent a rare and often overlooked cause of chronic medial knee pain. Their nonspecific clinical presentation frequently mimics more common pathologies, contributing to delayed diagnosis. This case underscores the importance of maintaining a broad differential diagnosis in patients with persistent medial knee symptoms and unremarkable physical examination findings. MRI plays a critical role in accurately identifying these lesions, delineating their anatomic relationships, and guiding surgical planning. When conservative management fails, arthroscopic excision provides effective symptom relief with a low risk of recurrence. Increased awareness of this uncommon entity may facilitate earlier diagnosis and appropriate intervention, ultimately improving patient outcomes.
